# An Overview of Computational Coronary Physiology Technologies Based on Medical Imaging and Artificial Intelligence

**DOI:** 10.31083/j.rcm2506211

**Published:** 2024-06-13

**Authors:** Bin Li, Huaigang Chen, Hong Wang, Lang Hong, Liu Yang

**Affiliations:** ^1^Department of Cardiology, Jiangxi Provincial People’s Hospital, The First Affiliated Hospital of Nanchang Medical College, 330006 Nanchang, Jiangxi, China; ^2^Jiangxi Medical College, Nanchang University, 330036 Nanchang, Jiangxi, China

**Keywords:** coronary hemodynamics, non-invasive assessment, medical imaging, quantitative flow ratio, optical flow ratio, computational fractional flow reserve, instantaneous wave-free ratio

## Abstract

This article reviews four new technologies for assessment of coronary hemodynamics based on medical imaging and artificial intelligence, including quantitative flow ratio (QFR), optical flow ratio (OFR), computational fractional flow reserve (CT-FFR) and artificial intelligence (AI)-based instantaneous wave-free ratio (iFR). These technologies use medical imaging such as coronary angiography, computed tomography angiography (CTA), and optical coherence tomography (OCT), to reconstruct three-dimensional vascular models through artificial intelligence algorithms, simulate and calculate hemodynamic parameters in the coronary arteries, and achieve non-invasive and rapid assessment of the functional significance of coronary stenosis. This article details the working principles, advantages such as non-invasiveness, efficiency, accuracy, limitations such as image dependency, and assumption restrictions, of each technology. It also compares and analyzes the image dependency, calculation accuracy, calculation speed, and operation simplicity, of the four technologies. The results show that these technologies are highly consistent with the traditional invasive wire method, and shows distinct advantages in terms of accuracy, reliability, convenience and cost-effectiveness, but there are also factors that affect accuracy. The results of this review demonstrates that AI-based iFR technology is currently one of the most promising technologies. The main challenges and directions for future development are also discussed. These technologies bring new ideas for the non-invasive assessment of coronary artery disease, and are expected to promote the technological progress in this field.

## 1. Introduction

Coronary artery disease (CAD) is caused by atherosclerosis-induced narrowing or occlusion of the coronary arteries, and it is one of the most common cardiovascular diseases worldwide [1], as well as a major cause of death and disability [2]. According to the World Health Organization (WHO), CAD causes about 9 million deaths annually, accounting for 16% of the global mortality [3]. An important aspect of the diagnosis and treatment of CAD is the assessment of coronary hemodynamics, such as pressure, flow and resistance in the coronary arteries, which is reflective of the functional status and blood perfusion of the coronary arteries. Assessment of computational hemodynamics is of great clinical value for determining the functional significance of coronary stenosis, guiding the strategy selection and outcome evaluation of percutaneous coronary intervention (PCI), and predicting the prognosis and risk of recurrence disease in patients.

Currently, the most commonly used method for computational hemodynamics assessment is the wire method, which involves inserting a pressure wire into the coronary artery, measuring the pressure gradient at different locations in the coronary artery, and calculating indicators such as fractional flow reserve (FFR) or instantaneous wave-free ratio (iFR). FFR is the ratio of pressure before and after a certain location in the coronary artery under maximal blood flow conditions (usually achieved by injecting vasodilators such as acetylcholine or nitroglycerin) [4]. iFR is the ratio of pressure before and after a certain location in the coronary artery during a specific period of the cardiac cycle (such as early or late diastole). The wire method has high accuracy and reliability, and has become the gold standard for assessment of computational hemodynamics [5]. However, the wire method also has some limitations, such as complexity, increased cost, invasiveness, and the need for vasodilators. To overcome the drawbacks of the wire method, computational hemodynamic technologies based on medical imaging and artificial intelligence have emerged in recent years. These technologies use imaging data (such as coronary angiography, computed tomography angiography (CTA), and optical coherence tomography (OCT)) to perform three-dimensional reconstruction and blood flow simulation (including image preprocessing, feature extraction, geometric reconstruction, and blood flow simulation), and thus can quickly and accurately calculate FFR or iFR without the need for wires or drugs. These technologies include quantitative flow ratio (QFR) [6], optical flow ratio (OFR) [7], computational fractional flow reserve (CT-FFR) [8], and artificial intelligence (AI)-based iFR [9]. These technologies not only improve the efficiency and convenience of computational hemodynamics assessment, but also reduce the cost and risk associated with these techniques.

The aim of this article is to review the principles, methods, advantages, disadvantages and clinical applications of these computational hemodynamic technologies based on medical imaging and artificial intelligence, and to compare and analyze them with the conventional wire method, in order to provide new perspectives and references for the diagnosis and treatment of CAD.

## 2. Computational Coronary Physiology Technologies

### 2.1 QFR Technology

**Fig. 1.**
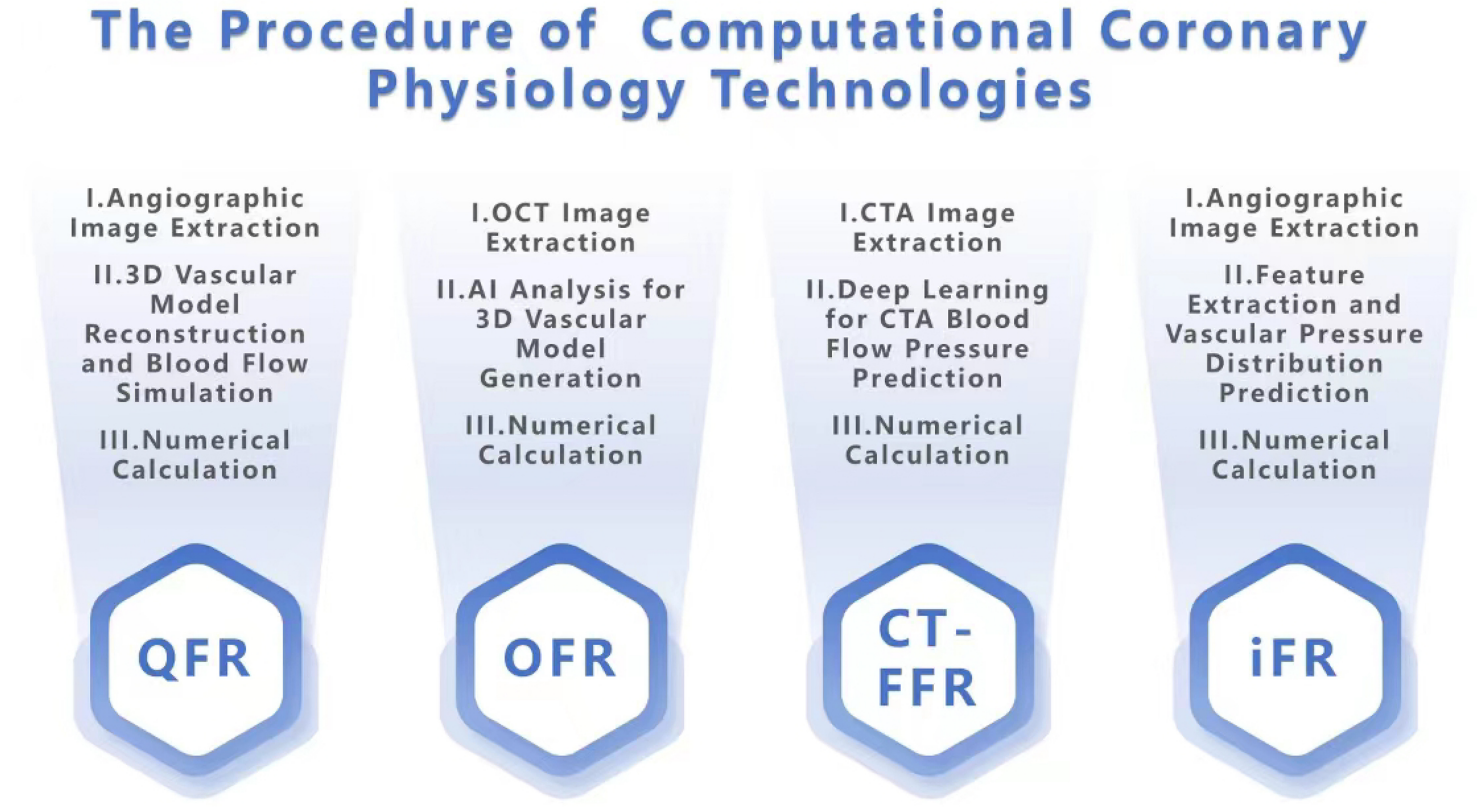
**Schematic diagram of the principles and steps of the four technologies. **QFR, quantitative flow ratio; OFR, optical flow ratio; CT-FFR, computational fractional flow reserve; OCT, optical coherence tomography; CTA, computed tomography angiography; iFR, instantaneous wave-free ratio; 3D, Three-Dimensional.

**Fig. 2.**
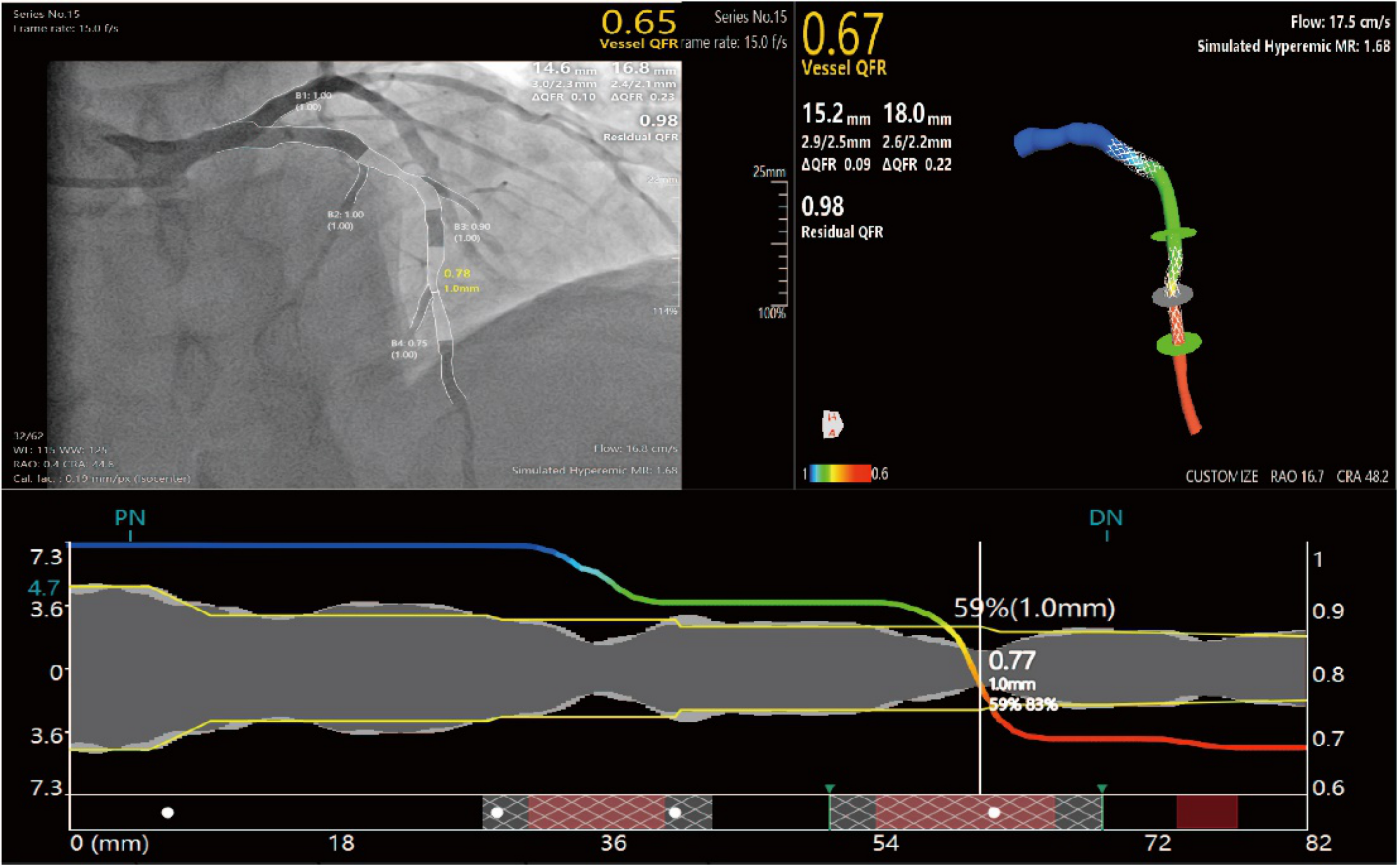
**Example of quantitative flow ratio (QFR) technology principle: coronary angiography image data and fractional flow reserve (FFR) indicator of an anonymous patient. **MR, microcirculatory resistance; RAO, right anterior oblique; CRA, cranial; PN represents the reference lumen proximal to the lesion; DN represents the reference lumen distal to the lesion.

QFR technology is a wire-free FFR rapid analysis system that uses artificial intelligence to reconstruct three-dimensional vascular models from coronary angiography images and simulate the calculation of FFR indicators. QFR technology does not require additional intervention materials and drugs, and can quickly and accurately assess the functional significance of a coronary stenosis, guiding PCI treatment [10]. The principle is shown in Fig. 1. QFR technology calculates the FFR indicators through the following steps: (1) Extract the vascular contours from the coronary angiography images (using a certain algorithm to reconstruct the three-dimensional model of the coronary artery from two angiographic images with an angle difference of more than 25 degrees, and calculate the pressure distribution and cardiac motion in the vessel according to the vessel morphology and blood flow velocity). (2) Use artificial intelligence algorithms to reconstruct the three-dimensional vascular model and blood flow boundary conditions. (3) Use computational fluid dynamics (computational hemodynamics) methods to simulate the hemodynamic process. (4) Calculate the pressure gradient and FFR indicators at different positions. The principle and application of QFR technology are demonstrated in the data from a patient with a coronary stenosis in Fig. 2. In this patient, the FFR indicator calculated by QFR technology was 0.67, which was close to the FFR indicator measured by the wire method of 0.65, indicating that QFR technology can effectively simulate coronary hemodynamics (See Fig. 2).

QFR technology, as a wire-free FFR rapid analysis system [11], has many advantages: (1) Non-Invasive Procedure: QFR’s reliance on angiographic images mitigates the need for invasive measures, thereby reducing patient risk and discomfort. (2) Rapid Computational Analysis: The technology’s capability to quickly process static images streamlines the diagnostic process, significantly saving time in clinical practice. (3) Accurate and reliable: QFR technology uses artificial intelligence algorithms to improve the accuracy and automation of image processing and blood flow simulation, reducing the impact of human error and subjective judgment, thus improving the accuracy and reliability of the assessment of computational hemodynamics. (4) Clinically effective: QFR technology has been validated in multiple clinical trials for its consistency and correlation with the wire method, as well as its guidance during PCI treatment for better patient selection leading to improved patient outcomes [12, 13, 14, 15, 16]. However, QFR technology also has some disadvantages and shortcomings: (1) Image Quality Dependency: The accuracy of QFR assessments relies heavily on the quality of angiographic images, with issues like blurring or distortion potentially impacting outcomes. (2) Computational Model Limitations: The assumptions inherent in QFR’s computational models, such as the one-dimensional Navier-Stokes equation, might not fully represent complex physiological conditions. (3) Insufficient validation: Although QFR technology has been validated for its effectiveness in some clinical trials, it still lacks more long-term follow-up and larger size studies, as well as comparative studies with other computational hemodynamics technologies, thus requiring further accumulation of evidence and experience to improve its clinical application and confidence level. (4) Due to its reliance on angiographic images from a limited number of projections, this can lead to potential inaccuracies in the reconstruction of vessel geometry, as the quality and angles of these projections are critical for accurate assessment. In addition, based on angiographic images, the flow and pressure conditions at the stenotic boundary were simulated using computational fluid dynamics which may be difficult to perform. The estimation of flow boundary conditions in QFR involves the use of angiographic images to create a virtual model of the coronary artery.

### 2.2 OFR Technology

OFR technology is a wire-free FFR technology that uses the high resolution and accuracy of OCT images, and reconstructs three-dimensional vascular models through artificial intelligence technology, simulating the calculation of FFR indicators [17]. While OFR technology independently does not require pressure wires, the associated OCT procedure is invasive and often utilizes an imaging catheter. Additionally, intra-coronary nitroglycerin may be used to enhance vessel imaging, which can quickly and accurately assess the functional significance of coronary stenosis to guide PCI therapy. The principle of OFR technology is shown in Fig. 1.

The advantages of OFR technology are: (1) Superior Imaging Resolution: OFR’s utilization of OCT provides exceptionally detailed images of coronary microstructures, enhancing diagnostic precision. (2) Efficiency and Minimal Invasiveness: The technology’s ability to quickly process OCT images while being less invasive offers significant advantages in patient management. (3) Fast and efficient: OFR technology only requires multiple cross-sectional images to complete the computational hemodynamic assessment. The whole process can be completed in a few minutes, and the results can be displayed in real time, thus saving time and resources, and improving work efficiency. (4) Clinically effective [18, 19, 20]: OFR technology has been validated in multiple clinical trials for its consistency and correlation with the wire method, as well as its guidance for PCI treatment selection and outcome evaluation, thus providing effective clinical reference for clinicians. There are, however, several disadvantages of OFR technology: (1) OCT Image Quality Reliance: The effectiveness of OFR is closely tied to the clarity and accuracy of OCT images. Suboptimal quality can potentially result in diagnostic inaccuracies. (2) Modeling Assumption Constraints: Similar to QFR, OFR’s blood flow simulations are based on certain assumptions that may not fully capture the complexity of coronary hemodynamics. (3) Insufficient validation: Although OFR technology has been validated for its effectiveness in some clinical trials, it still lacks more long-term follow-up and larger-sample studies, as well as comparative studies with other computational hemodynamics technologies, thus requiring further accumulation of evidence and experience to improve the level of confidence in its clinical application. (4) The size of the catheters restricts the range of vessel imaging, typically requiring one vessel to be imaged at a time. This approach can be time-consuming and may not provide a comprehensive view of the coronary arterial system in a single procedure. Similarly, in OFR, the flow boundary conditions are estimated by a detailed analysis of the vascular anatomy. The flow boundary conditions are based on the detailed anatomical data obtained from optical imaging techniques. This includes assessing the lumen area and plaque characteristics, as well as the hemodynamics near the boundaries of the imaged vascular segments.

### 2.3 Deep Learning-Based CT-FFR Technology

CT-FFR technology is a wire-free FFR technology that uses a deep learning model to extract features and predict pressure from coronary CTA images, and calculates the FFR indicator [21]. CT-FFR technology does not require wires or drugs, and quickly and accurately assesses the functional significance of coronary stenosis, guiding PCI treatment. The principle and method of CT-FFR technology are shown in Fig. 1.

The advantages of CT-FFR technology are: (1) High speed and accuracy: CT-FFR technology utilizes the high-speed computation and high-precision prediction of the deep learning model, and can complete the computational hemodynamics assessment in a short time, and has a high consistency and correlation with the wire method. (2) Versatility in Vascular Conditions: CT-FFR’s ability to adapt to diverse vascular morphologies enhances its applicability across a broad spectrum of coronary lesions. (3) Strong adaptability: CT-FFR technology utilizes the powerful learning and generalization capabilities of the deep learning model, and can adapt to different vascular morphologies, diameters, and lengths, that can be applied to a wider range of patient populations. (4) Clinically effective: CT-FFR technology has been validated in multiple clinical trials for its role for selecting patients for PCI treatment, evaluating the outcomes following PCI, and predicting the risk for re-stenosis and patient prognosis [22]. Deep learning not only plays a role in system construction, but it also serves a crucial function in image segmentation and processing, particularly in the analysis of cardiovascular imaging data. Deep learning technology enhances the precision of segmentation and optimizes processing workflows, thereby improving the accuracy and efficiency of CT-FFR assessments. This technology is particularly effective in the automated identification of complex cardiovascular structures, reducing the need for manual intervention, and enhancing overall diagnostic outcomes. The disadvantages of CT-FFR technology are: (1) CTA Image Dependency: The accuracy of CT-FFR is contingent upon the quality of coronary CTA images, with lower quality images potentially affecting the reliability of assessments. (2) Complexity in Model Development: The development and training of effective deep learning models for CT-FFR involve significant challenges, requiring extensive data and computational resources which may not be available in all hospital systems.

### 2.4 AI-Based iFR Technology

AI-based iFR technology is a wire-free FFR technology that utilizes an artificial intelligence model to extract features and predict iFR indicators from coronary angiography images [23]. AI-based iFR technology does not require wires or drugs, and rapidly and accurately assesses the functional significance of coronary stenosis, guiding PCI treatment. The principle and method of AI-based iFR technology are shown in Fig. 1.

The advantages of iFR technology are: High speed and accuracy: AI-based iFR technology utilizes the high-speed computation and high-precision prediction of the deep learning model, and can complete the computational hemodynamic assessment in a few seconds, and has a high consistency and correlation with the wire method. (1) Non-invasive or minimally invasive: AI-based iFR technology does not require the insertion of wires or the injection of drugs, and only requires routine coronary angiography to complete the computational hemodynamic assessment, thus avoiding the trauma, complications, and discomfort, caused by wires or drugs. (2) Wide Adaptability: The technology’s capacity to adjust to various imaging conditions and patient profiles enhances its utility in diverse clinical scenarios. (3) Clinically effective: AI-based iFR technology has been validated in multiple clinical trials for its role for PCI treatment selection and outcomes, as well as its predictive role for patient prognosis and the risk of recurrence risk [24, 25]. The disadvantages of iFR technology are: (1) Reliance on Angiographic Image Quality: The precision of iFR is dependent on the quality of angiographic images, with poor quality potentially leading to less reliable outcomes. (2) Demands of AI Model Training: iFR’s reliance on AI necessitates substantial training data and computational resources, posing challenges in model development and implementation. These technologies have their own indications and limitations in different clinical scenarios, thus requiring the selection of appropriate technologies according to the specific conditions of the patients and the judgment of the doctors. The transition to AI iFR and iFR techniques entails directly measuring intracoronary pressure to assess hemodynamic conditions at lesion boundaries. These methods diverge in their approach to estimate boundary conditions: AI iFR and iFR determine flow dynamics at lesion boundaries through intracoronary pressure gradients, while CT-based FFR utilizes CT angiography coupled with computational modeling to delineate flow and pressure along the coronary artery, thus defining the boundary conditions. In addition, the process requires high quality angiographic images. These standards include the need for high-resolution images with minimal motion blur, consistent and adequate contrast, and a clear view of the relevant coronary segment. These image quality parameters are critical as they directly impact the AI algorithm’s ability to accurately assess and calculate iFR values. The operator must ensure that these imaging criteria are met to facilitate the AI’s analytical process and to guarantee the reliability of the derived iFR estimations.

### 2.5 Indications and Clinical Applications of Coronary Imaging Technologies

OFR and QFR (technologies based on Cath Lab): Suitable for patients where real-time, detailed coronary artery imaging is crucial, such as in cases of complex coronary artery disease. Particularly useful in emergency situations, such as during angioplasty, where immediate assessment of the intervention is required. On the other hand, the deep learning-based CT-FFR technology leverages the capabilities of deep learning algorithms and is ideal for non-invasive and accurate estimation of fractional flow reserve. It is suitable for patients who require a comprehensive coronary artery assessment without the risks or discomfort of invasive procedures. iFR, is suitable for physiological assessment of coronary artery stenosis, particularly in situations where medications that cause congestion are contraindicated or undesirable.

### 2.6 Development and Trends of Functional Evaluation Techniques for Coronary Artery Stenosis in Global Guidelines and Expert Consensus 

The development and trends of these four techniques in global guidelines and expert consensus statements reflect their status and value in the diagnosis and treatment of coronary artery disease.

In 2019, the Fuwai Hospital of Chinese Academy of Medical Sciences and Cardiovascular Disease Branch of Chinese Medical Association released the Clinical Pathway of Coronary Artery CT Blood Flow Reserve Fraction Application China Expert Consensus [26], which introduced the principle, technology, workflow, clinical application, advantages and limitations of CT-FFR, as well as its comparison and combination with other techniques. The consensus gave the clinical indications, contraindications, operation steps, interpretation, report template, quality control and other contents of CT-FFR, as well as some common questions and answers.

In 2021, the American College of Cardiology (ACC) and the American Heart Association (AHA) released the 2021 Guidelines for Coronary Artery Interventional Therapy [27], which recommended FFR, iFR and CT-FFR as methods to assess the functional significance of coronary artery stenosis, to guide the decision for coronary revascularization. The guidelines gave the advantages and limitations, evidence level and recommendation level of different techniques, as well as the comparison and combination with other evaluation methods.

In 2021, The Lancet published the results of the FAVOR III China study initiated by the Fuwai Hospital of Chinese Academy of Medical Sciences, which was a large-scale randomized controlled clinical trial [28], aiming to evaluate whether QFR-guided PCI treatment was superior to conventional angiography-guided intervention treatment, whether it could improve the prognosis of patients, and reduce the economic burden. The study demonstrated that QFR-guided PCI could significantly reduce the risk of coronary PCI by 35%, while reducing medical costs. Furthermore, it is important to emphasize one of the main advantages of QFR—its demonstrated clinical benefits in large-scale randomized trials. These trials have provided robust evidence of the effectiveness of QFR in clinical practice, underscoring its significant value as a diagnostic tool in cardiovascular care.

In 2022, the Cardiovascular Disease Branch of Chinese Medical Association and the Editorial Board of Chinese Journal of Cardiovascular Diseases released the Chinese Guideline for Interventional Treatment of Coronary Artery Left Main Bifurcation Lesions [29], which recommended the functional evaluation of left main bifurcation lesions to guide the treatment strategy, including FFR, iFR and QFR techniques. The guideline gave provided the indications, methodology, interpretation criteria and complications of the different techniques, as well as the comparison with the anatomical evaluation.

2022 JCS Guidelines: Diagnosis and Treatment of Patients with Stable Coronary Artery Disease mentioned CT-FFR and CT-FFR as non-invasive methods to assess coronary artery stenosis, and QFR as a computational physiology technique based on coronary angiography [30]. These techniques can improve the efficacy and safety of coronary artery interventional therapy.

**Fig. 3.**
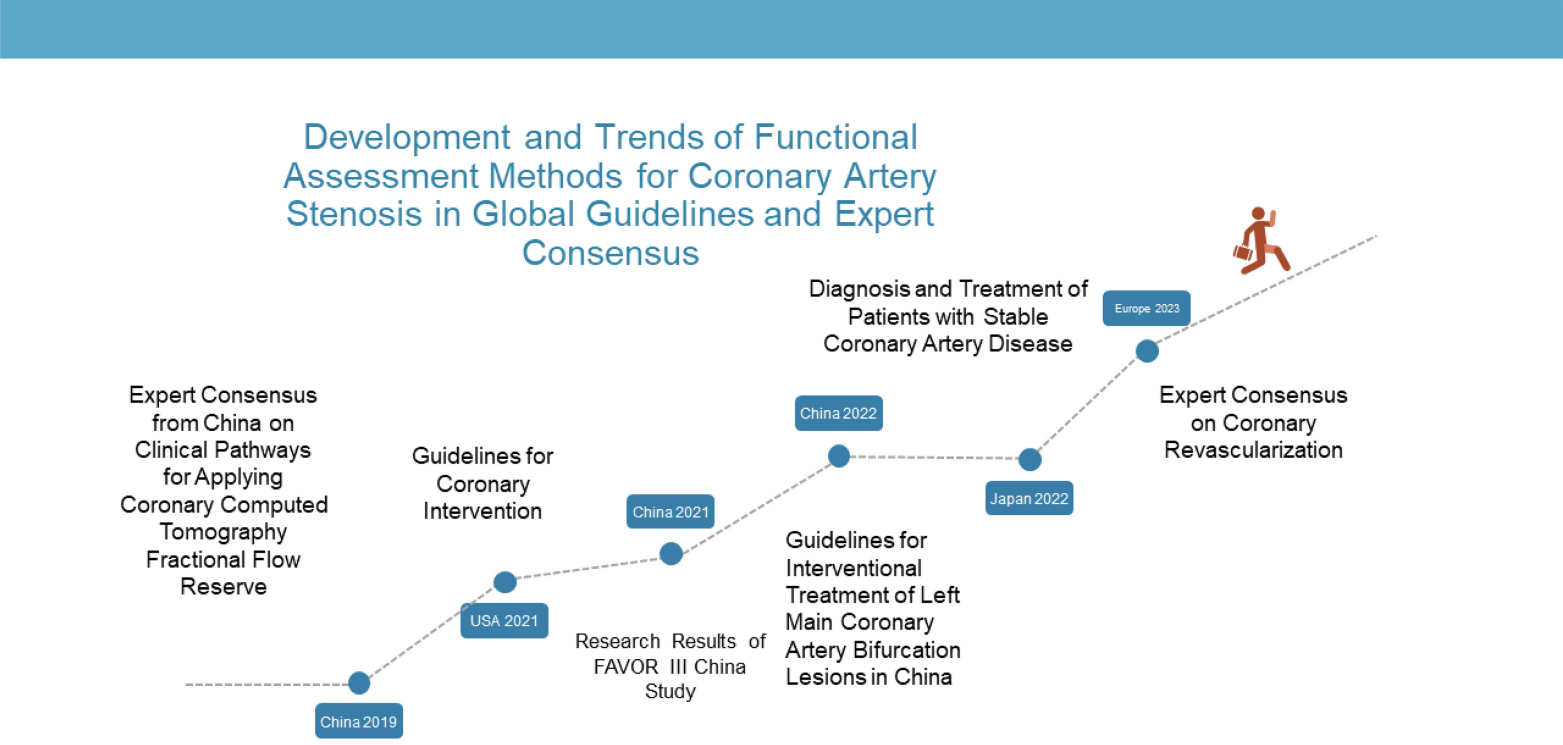
**Development and trends of functional evaluation techniques for coronary artery stenosis in global guidelines and expert consensus**.

In 2023, the European Heart Journal published an expert consensus on coronary revascularization based on cardiac CT [31], which mentioned cFFR and iFR as non-invasive methods to assess coronary artery stenosis, and QFR as a computational physiology technique based on coronary angiography. The consensus gave the advantages and disadvantages, scope of application and recommended thresholds of different techniques, as well as the comparison and consistency with invasive FFR. We drew a timeline chart, which showed the recommendation of functional evaluation techniques for coronary artery stenosis in the guidelines, clinical trials and expert consensus of different regions (China, USA, Japan and Europe) from 2019 to 2023. These techniques include the four techniques involved in this article. This chart can help to understand the development, history, and application of these techniques, as well as the comparisons between them (see Fig. 3).

## 3. Discussion: Comprehensive Evaluation of Computational Hemodynamic Techniques Based on Medical Imaging and Artificial Intelligence

This article introduced four computational hemodynamic techniques based on medical imaging and artificial intelligence, namely QFR, OFR, CT-FFR and AI-based iFR techniques, which are all representatives of wire-free FFR techniques. They can quickly and accurately assess the functional significance of coronary artery stenosis without the need for wires or drugs, and guide PCI treatment. This section will compare and analyze these four techniques in terms of image dependency, computational accuracy, computational speed and operational simplicity, in order to provide a reference for clinicians to choose the appropriate technique.

First, regarding image dependency, unlike CT-FFR which utilizes CT images for assessment, QFR employs angiographic images to evaluate the severity of coronary artery stenoses. OFR depends on OCT images, and iFR also uses angiographic images, each employing different imaging sources [32]. They use different algorithms. While CT-FFR is based on CT images, which are affected by heart rate, arrhythmia, motion artifacts, calcification, contrast agent concentration and other factors, QFR relies on coronary angiography images [33], which can cause the quality of CT images to decline, thus affecting the calculation of CT-FFR [34]. The article demonstrated that the diagnostic accuracy of CT MPI combined with CTA FFR was 79%, and the area under the curve increased from 0.78 to 0.85. On the other hand, OFR and iFR depend on OCT and angiographic images, respectively, which are affected by intravascular blood, and the need to perform blood removal or blood correction [35], which also increase the complexity and error of the calculation. Second, in evaluating computational accuracy, it is observed that CT-FFR and AI-based iFR show promising results, which may be attributed to the integration of advanced deep learning techniques [36]. However, it is important to recognize that the efficacy of technologies such as QFR and OFR could also be enhanced through similar advancements. The potential for accuracy improvements in various methodologies, including OCT or angio-based FFR systems, should not be overlooked, especially considering their high-resolution imaging capabilities. Compared with QFR and OFR, deep learning techniques can better deal with image noise, artifacts, calcification and other factors, can calculate FFR values faster, and can more accurately reflect the status of blood flow in blood vessels. However, some studies have also found that QFR and OFR have similar high consistency and correlation with FFR [37], while CT-FFR and AI-based iFR have some errors and variations, which may be related to the mismatch between the training data and validation data of the deep learning model [38]. In addition, regarding computational speed, while it’s often perceived that CT-FFR and iFR may offer quicker processing times through the use of Graphics Processing Unit (GPU)-accelerated deep learning models, it’s important to recognize that the speed is dependent on the specific implementation of each system. The computational efficiency is not solely determined by the imaging technique or the presence of GPU acceleration. This nuanced understanding is vital in comparing the processing speeds of QFR, OFR, CT-FFR, and iFR [39]. Deep learning models can perform a large number of matrix operations in parallel on GPUs, thereby improving the computational efficiency and accuracy [40]. However, some studies have also pointed out that the computational speed of QFR and OFR is also improving, and the results can be obtained within a few minutes by optimizing the algorithm and platform, and no additional equipment and software are required [41]. This requires the emergence of cheaper computing power. In terms of operational simplicity, QFR and iFR are better, the whole process is relatively simple, while OFR requires OCT imaging, and CT-FFR has higher requirements for computer equipment [42]. This is because CT-FFR needs to extract the geometric information and blood flow information of blood vessels from CT images, and then use complex computational fluid dynamics (computational hemodynamics) models and algorithms to calculate FFR values, which require high-performance computer hardware and software support. In addition, CTA more easily allows for a Three-Dimensional (3D) representation of the entire coronary tree in comparison to Angio or OCT, wherein individual vessels may be more easily segmented instead of the full tree due to the limited projections and slices. QFR and OFR are both techniques based on blood flow simulation, which require assumptions such as vessel wall thickness, blood viscosity, vessel elasticity and other parameters, which may vary greatly in different patients and different vessels, thus affecting the calculated results of FFR. On the contrary, some studies have also shown that the model assumptions of QFR and OFR do not affect their adaptability, as long as the corresponding blood flow parameters and boundary conditions are adjusted according to different blood flow conditions and vessel characteristics, accurate results can be obtained [43]. Finally, in terms of consistency, all four techniques are good, with a high degree of automation, stable and repeatable results [44, 45, 46]. This has also been confirmed by other studies, demonstrating that the results of the four techniques have high reproducibility with the results of FFR, and are not affected by the operator’s experience and skills. In addition to the above comparison, OFR has better interpretability because it combines accurate vascular structural information, while the interpretability of deep learning models is poor [47]. Cost-effectiveness refers to the comparison of the cost and resources required by the evaluation method with the clinical benefits and value it brings. Generally speaking, the higher the cost-effectiveness, the more the evaluation method can decrease medical costs and improve medical quality. QFR and iFR have higher portability and cost-effectiveness, because they only need conventional angiographic images (XA), do not need to inject vasodilators or use additional wires, operate simply and quickly, and have relatively low costs [48]. On the contrary, CT-FFR and OFR have lower portability and cost-effectiveness, because they need to use special equipment and images, such as CT or OCT, need to inject vasodilators or use additional wires, are more complex and operate slowly, and have relatively high costs [49] (See Table 1).

**Table 1.** **Comparative analysis of computational hemodynamics techniques based on medical imaging and artificial intelligence**.

Assessment method	QFR	CT-FFR	OFR	iFR
Image dependency	4	4	4	1
Calculation accuracy	5	5	3	5
Calculation speed	5	5	1	5
Ease of use	4	2	2	4
Adaptability	4	4	2	4
Repeatability	4	4	4	4
Explanatory	3	2	4	3
Portability	4	2	2	4
Cost effectiveness	5	2	2	5

QFR, quantitative flow ratio; OFR, optical flow ratio; CT-FFR, computational fractional flow reserve; iFR, instantaneous wave-free ratio. Table Note: This table uses a 1-to-5 rating scale for evaluation, where 1 represents the lowest and 5 represents the highest. For example, in the item of ‘image dependency’, 1 means the highest dependency on images, and 5 means the lowest.

## 4. Conclusion and Prospect

The main conclusion of this article is that the four techniques have their own advantages and disadvantages, and clinicians need to consider various factors such as accuracy, reliability, convenience and cost-effectiveness when choosing the appropriate technique, and make trade-offs and decisions according to the specific needs of the patient and their own experience and preference. We believe that the AI-based iFR technique is one of the most promising current techniques, which has high accuracy, reliability, convenience and cost-effectiveness, can adapt to different patient situations, can be combined with other techniques, and can improve the efficacy and safety of PCI therapy. In the future, the four techniques still have room for improvement and development, with the following points: (1) Further optimize and validate the deep learning model, improve its accuracy and reliability, solve the problem of its interpretability, and enhance its credibility and acceptability. (2) Further explore and compare the applicability and sensitivity of the four techniques in different types of and degrees of coronary artery stenosis, as well as their effectiveness and safety in different clinical scenarios, such as CT-FFR using machine learning and CAD reporting and data system (CAD-RADS) classification, and safely identifying significant coronary artery disease based on quantitative coronary angiography of patients before transcatheter aortic valve replacement [50]. (3) Further carry out large-scale randomized controlled clinical trials, evaluate the impact and value of the four techniques on PCI treatment, as well as the impact on the prognosis and quality of life of patients. (4) Further explore and utilize the complementarity and comprehensiveness of the four techniques, achieve multimodal coronary artery functional evaluation, and improve its comprehensiveness and accuracy. (5) The further use various models to explore the impact of functional evaluation of these techniques on the economic burden of patients, such as the long-term cost-effectiveness ratio based on Markov model or decision tree analysis.

## Data Availability

The datasets generated or analyzed during the study are available from the corresponding author on reasonable request.
